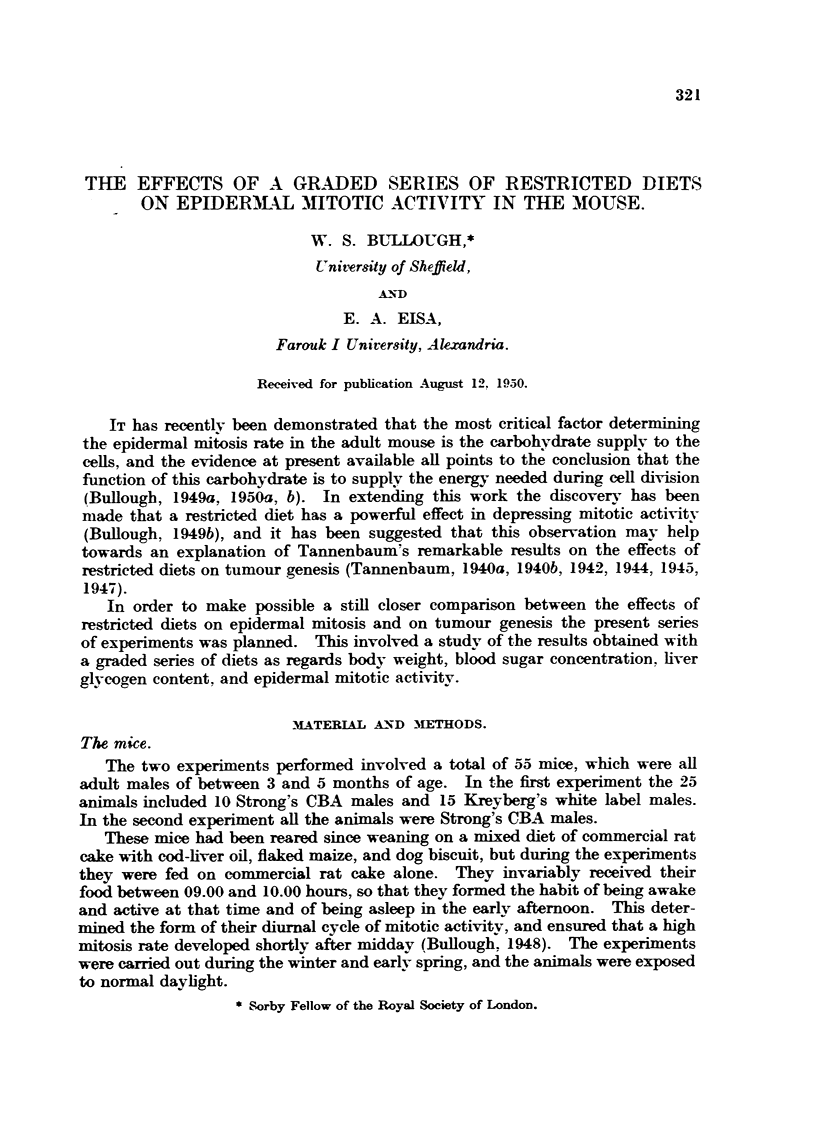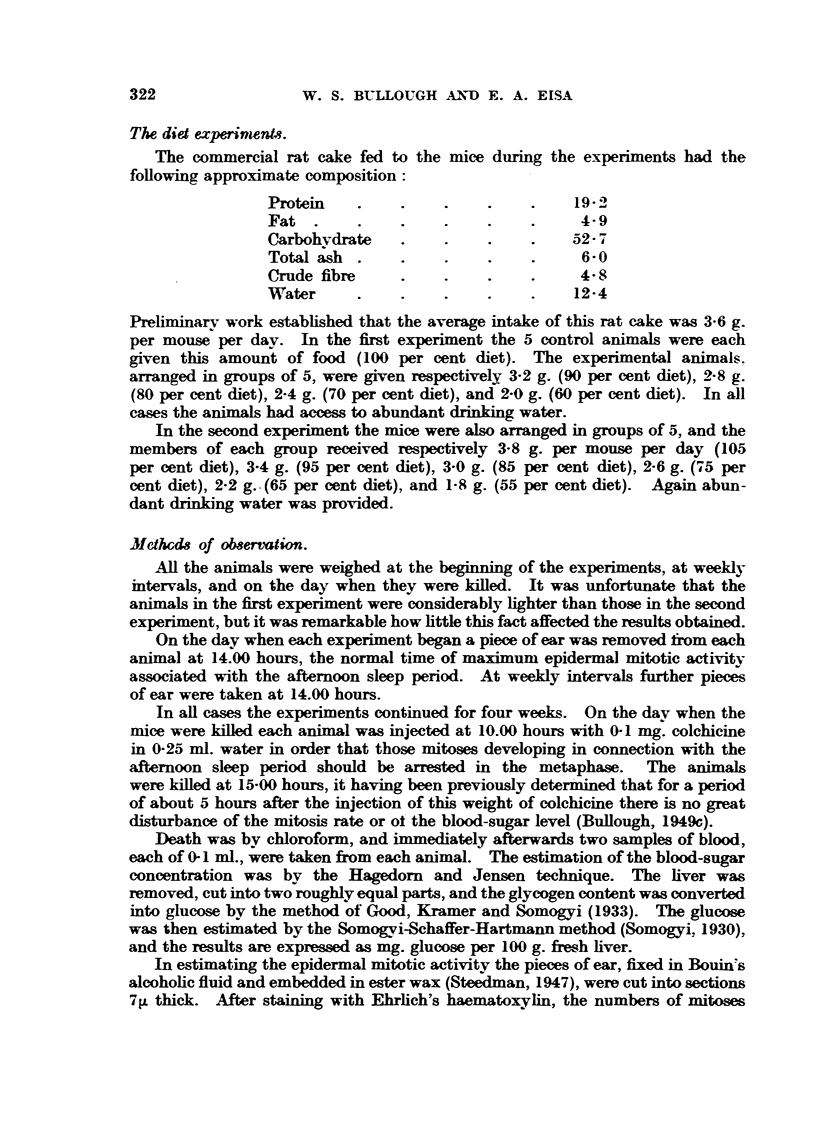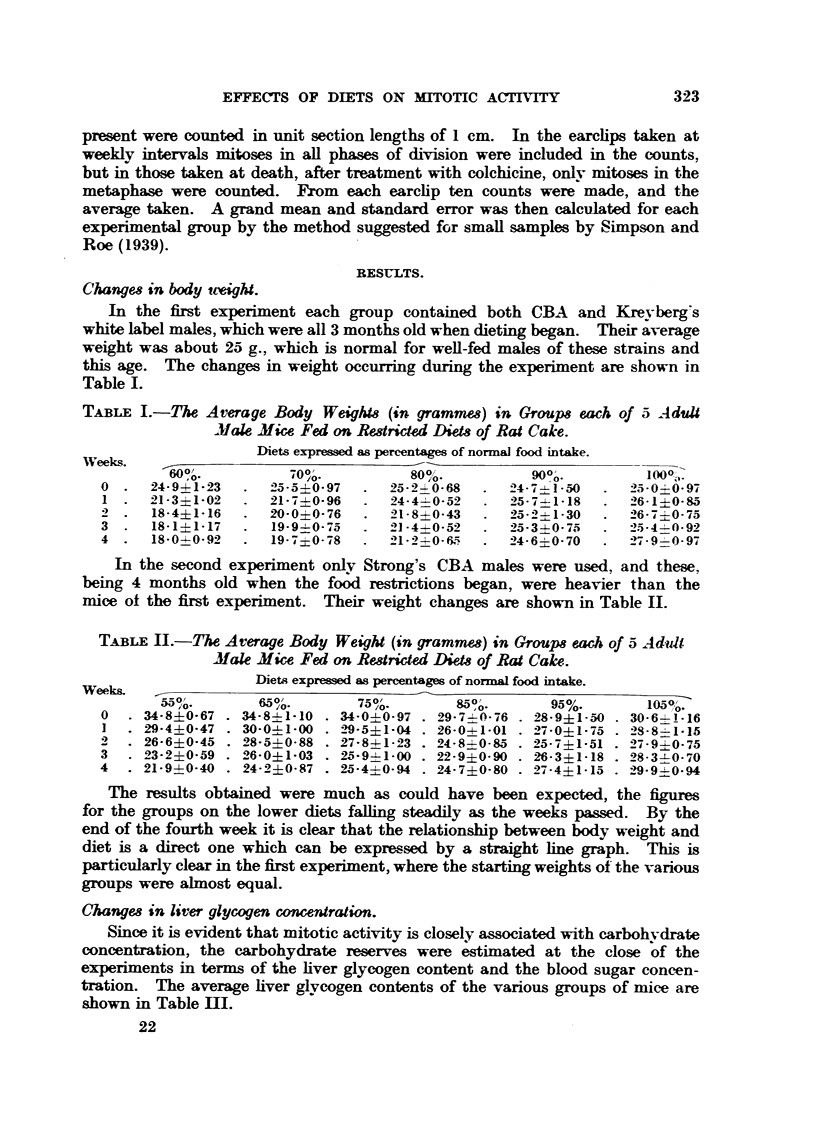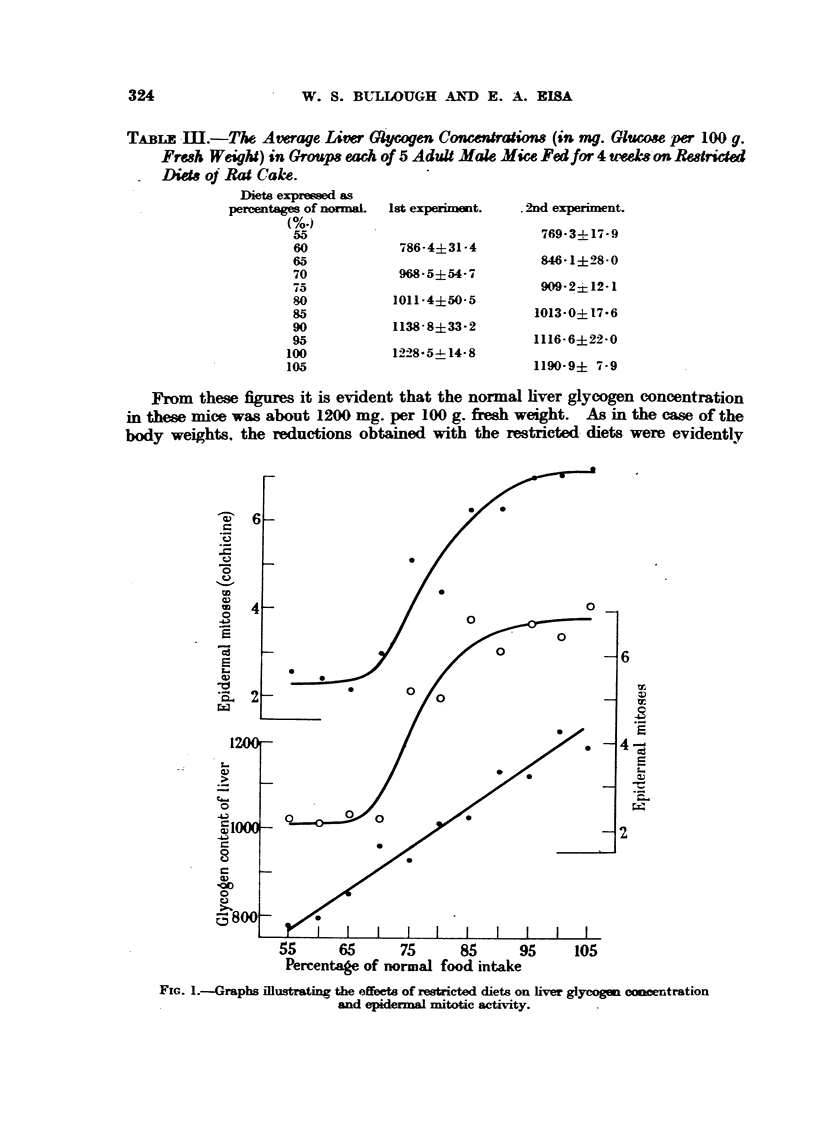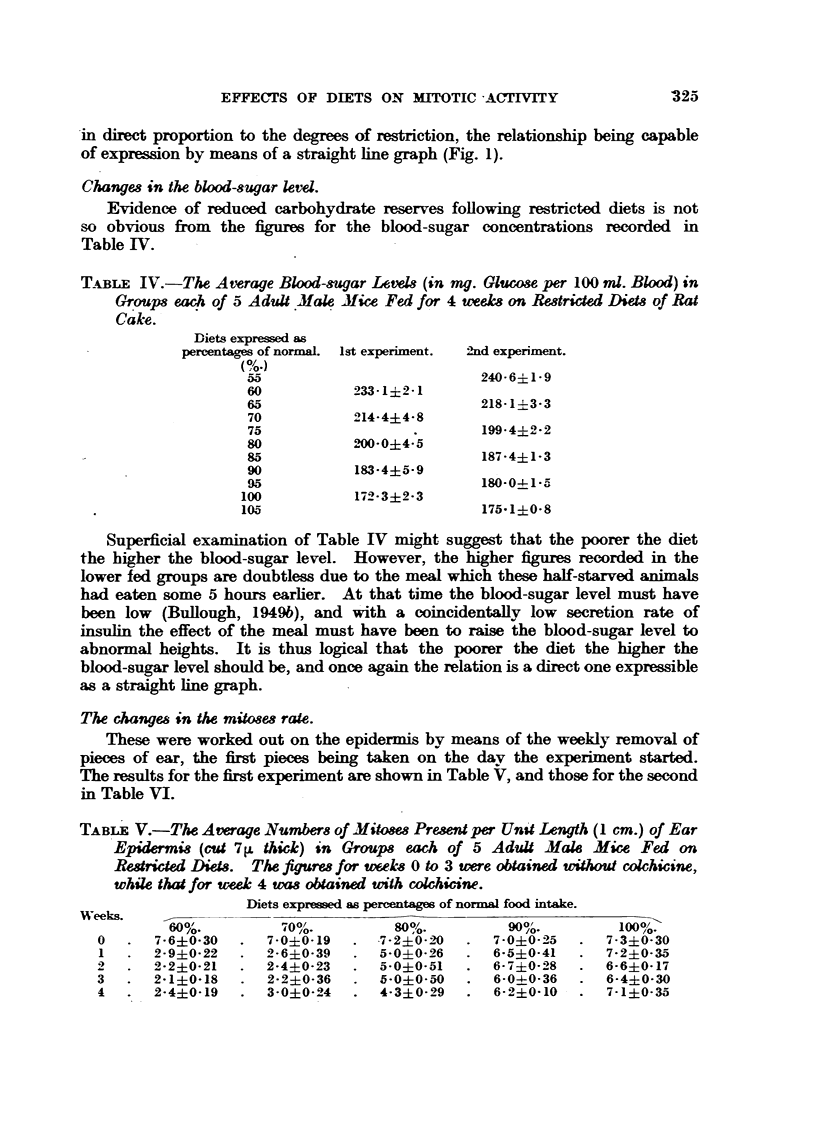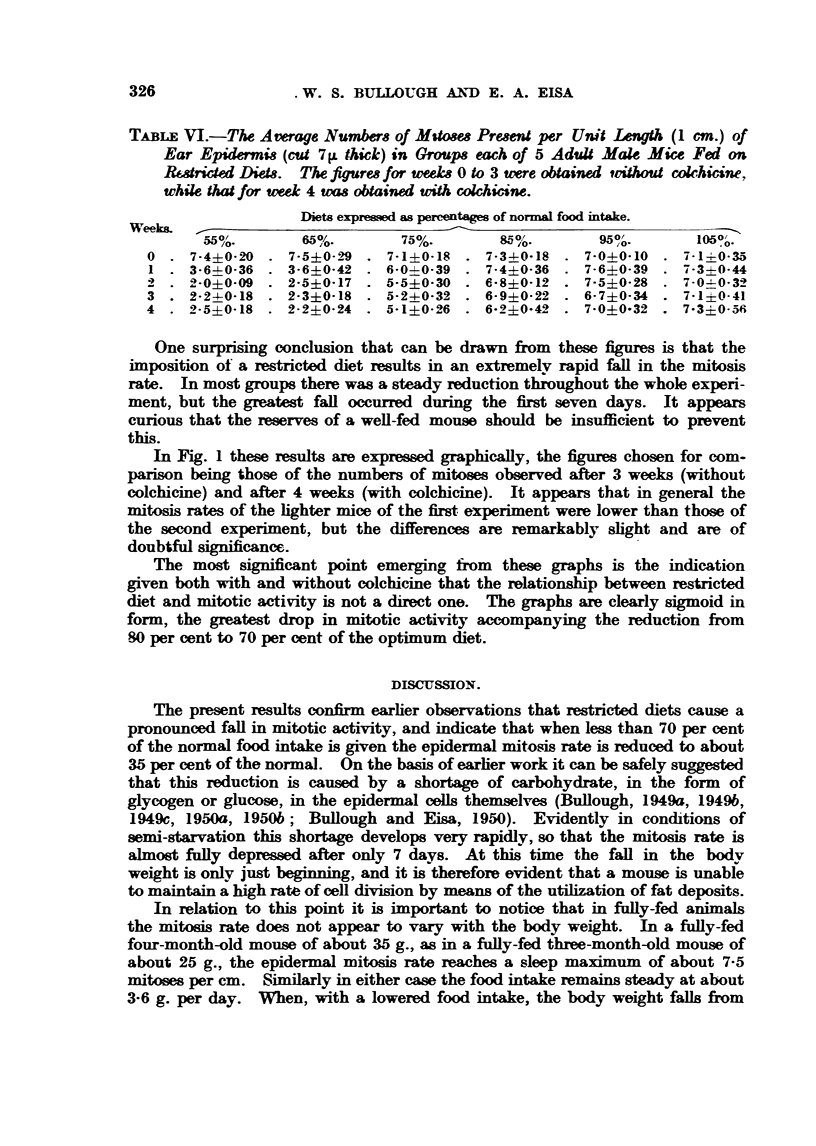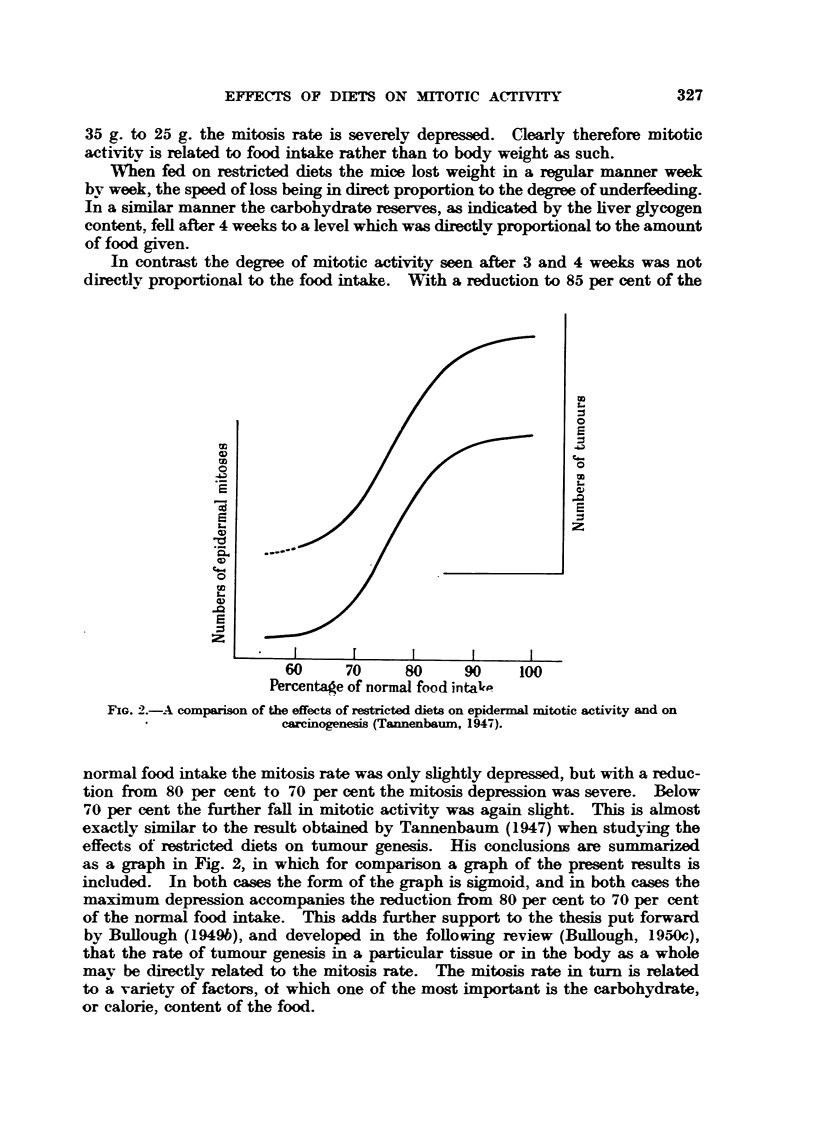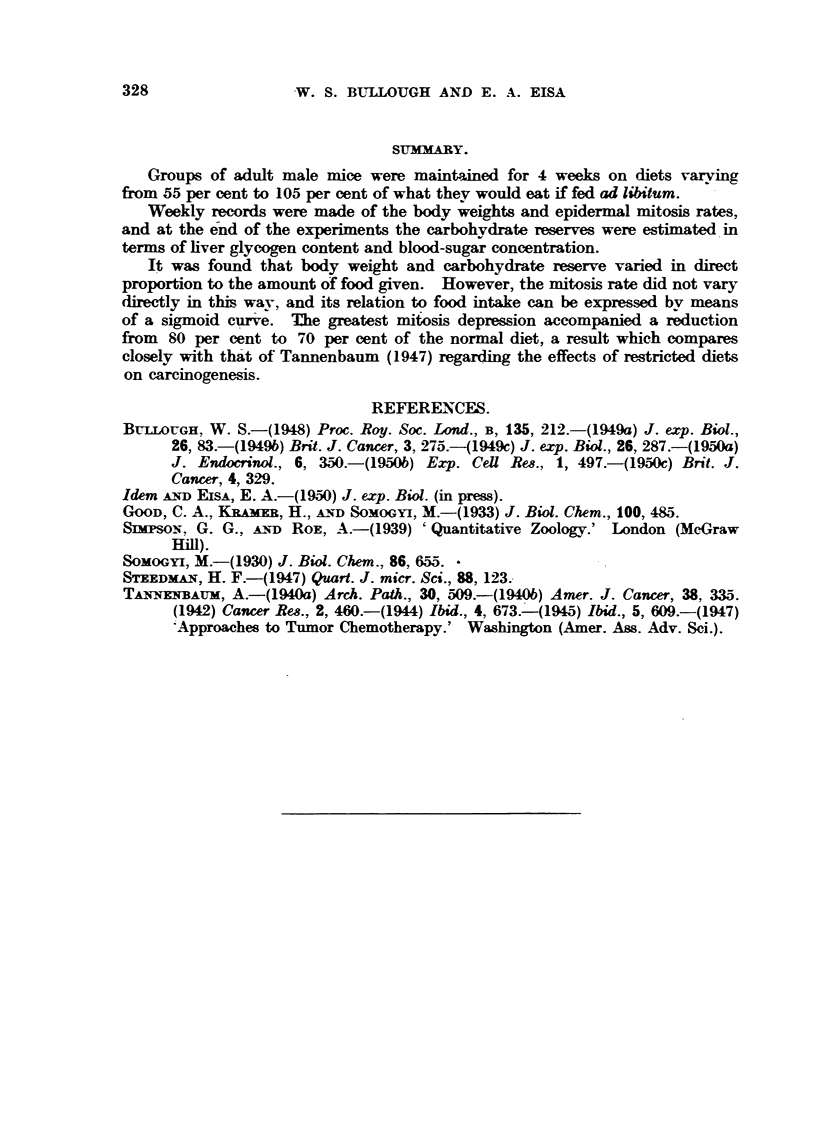# The Effects of a Graded Series of Restricted Diets on Epidermal Mitotic Activity in the Mouse

**DOI:** 10.1038/bjc.1950.30

**Published:** 1950-09

**Authors:** W. S. Bullough, E. A. Eisa


### THE EFFECTS OF A GRADED SERIES OF RESTRICTED DIETS ON EPIDERMAL MITOTIC ACTIVITY IN THE MOUSE.

W. S. BULLOUGH,*

*University of Sheffield,*

AND

E. A. EISA,

*Farouk I University, Alexandria.*

Received for publication August 12, 1950.

IT has recently been demonstrated that the most critical factor determining the epidermal mitosis rate in the adult mouse is the carbohydrate supply to the cells, and the evidence at present available all points to the conclusion that the function of this carbohydrate is to supply the energy needed during cell division (Bullough, 1949*a*, 1950*a*, *b*). In extending this work the discovery has been made that a restricted diet has a powerful effect in depressing mitotic activity (Bullough, 1949*b*), and it has been suggested that this observation may help towards an explanation of Tannenbaum's remarkable results on the effects of restricted diets on tumour genesis (Tannenbaum, 1940*a*, 1940*b*, 1942, 1944, 1945, 1947).

In order to make possible a still closer comparison between the effects of restricted diets on epidermal mitosis and on tumour genesis the present series of experiments was planned. This involved a study of the results obtained with a graded series of diets as regards body weight, blood sugar concentration, liver glycogen content, and epidermal mitotic activity.

### MATERIAL AND METHODS.

### *The mice.*

The two experiments performed involved a total of 55 mice, which were all adult males of between 3 and 5 months of age. In the first experiment the 25 animals included 10 Strong's CBA males and 15 Kreyberg's white label males. In the second experiment all the animals were Strong's CBA males.

These mice had been reared since weaning on a mixed diet of commercial rat cake with cod-liver oil, flaked maize, and dog biscuit, but during the experiments they were fed on commercial rat cake alone. They invariably received their food between 09.00 and 10.00 hours, so that they formed the habit of being awake and active at that time and of being asleep in the early afternoon. This determined the form of their diurnal cycle of mitotic activity, and ensured that a high mitosis rate developed shortly after midday (Bullough, 1948). The experiments were carried out during the winter and early spring, and the animals were exposed to normal daylight.

* Sorby Fellow of the Royal Society of London.

*The diet experiments.*

The commercial rat cake fed to the mice during the experiments had the following approximate composition :

| | |
|---|---|
| Protein . . . . . | 19·2 |
| Fat . . . . . . . | 4·9 |
| Carbohydrate . . . . | 52·7 |
| Total ash . . . . . | 6·0 |
| Crude fibre . . . . | 4·8 |
| Water . . . . . | 12·4 |

Preliminary work established that the average intake of this rat cake was 3·6 g. per mouse per day. In the first experiment the 5 control animals were each given this amount of food (100 per cent diet). The experimental animals, arranged in groups of 5, were given respectively 3·2 g. (90 per cent diet), 2·8 g. (80 per cent diet), 2·4 g. (70 per cent diet), and 2·0 g. (60 per cent diet). In all cases the animals had access to abundant drinking water.

In the second experiment the mice were also arranged in groups of 5, and the members of each group received respectively 3·8 g. per mouse per day (105 per cent diet), 3·4 g. (95 per cent diet), 3·0 g. (85 per cent diet), 2·6 g. (75 per cent diet), 2·2 g. (65 per cent diet), and 1·8 g. (55 per cent diet). Again abundant drinking water was provided.

*Methods of observation.*

All the animals were weighed at the beginning of the experiments, at weekly intervals, and on the day when they were killed. It was unfortunate that the animals in the first experiment were considerably lighter than those in the second experiment, but it was remarkable how little this fact affected the results obtained.

On the day when each experiment began a piece of ear was removed from each animal at 14.00 hours, the normal time of maximum epidermal mitotic activity associated with the afternoon sleep period. At weekly intervals further pieces of ear were taken at 14.00 hours.

In all cases the experiments continued for four weeks. On the day when the mice were killed each animal was injected at 10.00 hours with 0·1 mg. colchicine in 0·25 ml. water in order that those mitoses developing in connection with the afternoon sleep period should be arrested in the metaphase. The animals were killed at 15·00 hours, it having been previously determined that for a period of about 5 hours after the injection of this weight of colchicine there is no great disturbance of the mitosis rate or of the blood-sugar level (Bullough, 1949c).

Death was by chloroform, and immediately afterwards two samples of blood, each of 0·1 ml., were taken from each animal. The estimation of the blood-sugar concentration was by the Hagedorn and Jensen technique. The liver was removed, cut into two roughly equal parts, and the glycogen content was converted into glucose by the method of Good, Kramer and Somogyi (1933). The glucose was then estimated by the Somogyi-Schaffer-Hartmann method (Somogyi, 1930), and the results are expressed as mg. glucose per 100 g. fresh liver.

In estimating the epidermal mitotic activity the pieces of ear, fixed in Bouin's alcoholic fluid and embedded in ester wax (Steedman, 1947), were cut into sections 7$\mu$ thick. After staining with Ehrlich's haematoxylin, the numbers of mitoses present were counted in unit section lengths of 1 cm. In the earclips taken at weekly intervals mitoses in all phases of division were included in the counts, but in those taken at death, after treatment with colchicine, only mitoses in the metaphase were counted. From each earclip ten counts were made, and the average taken. A grand mean and standard error was then calculated for each experimental group by the method suggested for small samples by Simpson and Roe (1939).

### RESULTS.

### *Changes in body weight.*

In the first experiment each group contained both CBA and Kreyberg's white label males, which were all 3 months old when dieting began. Their average weight was about 25 g., which is normal for well-fed males of these strains and this age. The changes in weight occurring during the experiment are shown in Table I.

TABLE I.—*The Average Body Weights (in grammes) in Groups each of 5 Adult Male Mice Fed on Restricted Diets of Rat Cake.*

| Weeks. | Diets expressed as percentages of normal food intake. | | | | |
|---|---|---|---|---|---|
| | 60%. | 70%. | 80%. | 90%. | 100%. |
| 0 | 24·9±1·23 | 25·5±0·97 | 25·2±0·68 | 24·7±1·50 | 25·0±0·97 |
| 1 | 21·3±1·02 | 21·7±0·96 | 24·4±0·52 | 25·7±1·18 | 26·1±0·85 |
| 2 | 18·4±1·16 | 20·0±0·76 | 21·8±0·43 | 25·2±1·30 | 26·7±0·75 |
| 3 | 18·1±1·17 | 19·9±0·75 | 21·4±0·52 | 25·3±0·75 | 25·4±0·92 |
| 4 | 18·0±0·92 | 19·7±0·78 | 21·2±0·65 | 24·6±0·70 | 27·9±0·97 |

In the second experiment only Strong's CBA males were used, and these, being 4 months old when the food restrictions began, were heavier than the mice of the first experiment. Their weight changes are shown in Table II.

TABLE II.—*The Average Body Weight (in grammes) in Groups each of 5 Adult Male Mice Fed on Restricted Diets of Rat Cake.*

| Weeks. | Diets expressed as percentages of normal food intake. | | | | | |
|---|---|---|---|---|---|---|
| | 55%. | 65%. | 75%. | 85%. | 95%. | 105%. |
| 0 | 34·8±0·67 | 34·8±1·10 | 34·0±0·97 | 29·7±0·76 | 28·9±1·50 | 30·6±1·16 |
| 1 | 29·4±0·47 | 30·0±1·00 | 29·5±1·04 | 26·0±1·01 | 27·0±1·75 | 28·8±1·15 |
| 2 | 26·6±0·45 | 28·5±0·88 | 27·8±1·23 | 24·8±0·85 | 25·7±1·51 | 27·9±0·75 |
| 3 | 23·2±0·59 | 26·0±1·03 | 25·9±1·00 | 22·9±0·90 | 26·3±1·18 | 28·3±0·70 |
| 4 | 21·9±0·40 | 24·2±0·87 | 25·4±0·94 | 24·7±0·80 | 27·4±1·15 | 29·9±0·94 |

The results obtained were much as could have been expected, the figures for the groups on the lower diets falling steadily as the weeks passed. By the end of the fourth week it is clear that the relationship between body weight and diet is a direct one which can be expressed by a straight line graph. This is particularly clear in the first experiment, where the starting weights of the various groups were almost equal.

### *Changes in liver glycogen concentration.*

Since it is evident that mitotic activity is closely associated with carbohydrate concentration, the carbohydrate reserves were estimated at the close of the experiments in terms of the liver glycogen content and the blood sugar concentration. The average liver glycogen contents of the various groups of mice are shown in Table III.

TABLE III.—*The Average Liver Glycogen Concentrations (in mg. Glucose per 100 g. Fresh Weight) in Groups each of 5 Adult Male Mice Fed for 4 weeks on Restricted Diets of Rat Cake.*

| Diets expressed as percentages of normal. (%.) | 1st experiment. | 2nd experiment. |
|---|---|---|
| 55 | | 769·3±17·9 |
| 60 | 786·4±31·4 | |
| 65 | | 846·1±28·0 |
| 70 | 968·5±54·7 | |
| 75 | | 909·2±12·1 |
| 80 | 1011·4±50·5 | |
| 85 | | 1013·0±17·6 |
| 90 | 1138·8±33·2 | |
| 95 | | 1116·6±22·0 |
| 100 | 1228·5±14·8 | |
| 105 | | 1190·9± 7·9 |

From these figures it is evident that the normal liver glycogen concentration in these mice was about 1200 mg. per 100 g. fresh weight. As in the case of the body weights, the reductions obtained with the restricted diets were evidently

FIG. 1.—Graphs illustrating the effects of restricted diets on liver glycogen concentration and epidermal mitotic activity.

in direct proportion to the degrees of restriction, the relationship being capable of expression by means of a straight line graph (Fig. 1).

*Changes in the blood-sugar level.*

Evidence of reduced carbohydrate reserves following restricted diets is not so obvious from the figures for the blood-sugar concentrations recorded in Table IV.

TABLE IV.—*The Average Blood-sugar Levels (in mg. Glucose per 100 ml. Blood) in Groups each of 5 Adult Male Mice Fed for 4 weeks on Restricted Diets of Rat Cake.*

| Diets expressed as percentages of normal. (%.) | 1st experiment. | 2nd experiment. |
|---|---|---|
| 55 | | 240·6±1·9 |
| 60 | 233·1±2·1 | |
| 65 | | 218·1±3·3 |
| 70 | 214·4±4·8 | |
| 75 | | 199·4±2·2 |
| 80 | 200·0±4·5 | |
| 85 | | 187·4±1·3 |
| 90 | 183·4±5·9 | |
| 95 | | 180·0±1·5 |
| 100 | 172·3±2·3 | |
| 105 | | 175·1±0·8 |

Superficial examination of Table IV might suggest that the poorer the diet the higher the blood-sugar level. However, the higher figures recorded in the lower fed groups are doubtless due to the meal which these half-starved animals had eaten some 5 hours earlier. At that time the blood-sugar level must have been low (Bullough, 1949*b*), and with a coincidentally low secretion rate of insulin the effect of the meal must have been to raise the blood-sugar level to abnormal heights. It is thus logical that the poorer the diet the higher the blood-sugar level should be, and once again the relation is a direct one expressible as a straight line graph.

*The changes in the mitoses rate.*

These were worked out on the epidermis by means of the weekly removal of pieces of ear, the first pieces being taken on the day the experiment started. The results for the first experiment are shown in Table V, and those for the second in Table VI.

TABLE V.—*The Average Numbers of Mitoses Present per Unit Length (1 cm.) of Ear Epidermis (cut 7μ thick) in Groups each of 5 Adult Male Mice Fed on Restricted Diets. The figures for weeks 0 to 3 were obtained without colchicine, while that for week 4 was obtained with colchicine.*

| Weeks. | Diets expressed as percentages of normal food intake. | | | | |
|---|---|---|---|---|---|
| | 60%. | 70%. | 80%. | 90%. | 100%. |
| 0 | 7·6±0·30 | 7·0±0·19 | 7·2±0·20 | 7·0±0·25 | 7·3±0·30 |
| 1 | 2·9±0·22 | 2·6±0·39 | 5·0±0·26 | 6·5±0·41 | 7·2±0·35 |
| 2 | 2·2±0·21 | 2·4±0·23 | 5·0±0·51 | 6·7±0·28 | 6·6±0·17 |
| 3 | 2·1±0·18 | 2·2±0·36 | 5·0±0·50 | 6·0±0·36 | 6·4±0·30 |
| 4 | 2·4±0·19 | 3·0±0·24 | 4·3±0·29 | 6·2±0·10 | 7·1±0·35 |

TABLE VI.—*The Average Numbers of Mitoses Present per Unit Length* (1 *cm.*) *of Ear Epidermis* (*cut* 7 $\mu$ *thick*) *in Groups each of* 5 *Adult Male Mice Fed on Restricted Diets. The figures for weeks* 0 *to* 3 *were obtained without colchicine, while that for week* 4 *was obtained with colchicine.*

| Weeks. | Diets expressed as percentages of normal food intake. | | | | | |
|---|---|---|---|---|---|---|
| | 55%. | 65%. | 75%. | 85%. | 95%. | 105%. |
| 0 | 7·4±0·20 | 7·5±0·29 | 7·1±0·18 | 7·3±0·18 | 7·0±0·10 | 7·1±0·35 |
| 1 | 3·6±0·36 | 3·6±0·42 | 6·0±0·39 | 7·4±0·36 | 7·6±0·39 | 7·3±0·44 |
| 2 | 2·0±0·09 | 2·5±0·17 | 5·5±0·30 | 6·8±0·12 | 7·5±0·28 | 7·0±0·32 |
| 3 | 2·2±0·18 | 2·3±0·18 | 5·2±0·32 | 6·9±0·22 | 6·7±0·34 | 7·1±0·41 |
| 4 | 2·5±0·18 | 2·2±0·24 | 5·1±0·26 | 6·2±0·42 | 7·0±0·32 | 7·3±0·56 |

One surprising conclusion that can be drawn from these figures is that the imposition of a restricted diet results in an extremely rapid fall in the mitosis rate. In most groups there was a steady reduction throughout the whole experiment, but the greatest fall occurred during the first seven days. It appears curious that the reserves of a well-fed mouse should be insufficient to prevent this.

In Fig. 1 these results are expressed graphically, the figures chosen for comparison being those of the numbers of mitoses observed after 3 weeks (without colchicine) and after 4 weeks (with colchicine). It appears that in general the mitosis rates of the lighter mice of the first experiment were lower than those of the second experiment, but the differences are remarkably slight and are of doubtful significance.

The most significant point emerging from these graphs is the indication given both with and without colchicine that the relationship between restricted diet and mitotic activity is not a direct one. The graphs are clearly sigmoid in form, the greatest drop in mitotic activity accompanying the reduction from 80 per cent to 70 per cent of the optimum diet.

### DISCUSSION.

The present results confirm earlier observations that restricted diets cause a pronounced fall in mitotic activity, and indicate that when less than 70 per cent of the normal food intake is given the epidermal mitosis rate is reduced to about 35 per cent of the normal. On the basis of earlier work it can be safely suggested that this reduction is caused by a shortage of carbohydrate, in the form of glycogen or glucose, in the epidermal cells themselves (Bullough, 1949*a*, 1949*b*, 1949*c*, 1950*a*, 1950*b*; Bullough and Eisa, 1950). Evidently in conditions of semi-starvation this shortage develops very rapidly, so that the mitosis rate is almost fully depressed after only 7 days. At this time the fall in the body weight is only just beginning, and it is therefore evident that a mouse is unable to maintain a high rate of cell division by means of the utilization of fat deposits.

In relation to this point it is important to notice that in fully-fed animals the mitosis rate does not appear to vary with the body weight. In a fully-fed four-month-old mouse of about 35 g., as in a fully-fed three-month-old mouse of about 25 g., the epidermal mitosis rate reaches a sleep maximum of about 7·5 mitoses per cm. Similarly in either case the food intake remains steady at about 3·6 g. per day. When, with a lowered food intake, the body weight falls from

35 g. to 25 g. the mitosis rate is severely depressed. Clearly therefore mitotic activity is related to food intake rather than to body weight as such.

When fed on restricted diets the mice lost weight in a regular manner week by week, the speed of loss being in direct proportion to the degree of underfeeding. In a similar manner the carbohydrate reserves, as indicated by the liver glycogen content, fell after 4 weeks to a level which was directly proportional to the amount of food given.

In contrast the degree of mitotic activity seen after 3 and 4 weeks was not directly proportional to the food intake. With a reduction to 85 per cent of the

FIG. 2.—A comparison of the effects of restricted diets on epidermal mitotic activity and on carcinogenesis (Tannenbaum, 1947).

normal food intake the mitosis rate was only slightly depressed, but with a reduction from 80 per cent to 70 per cent the mitosis depression was severe. Below 70 per cent the further fall in mitotic activity was again slight. This is almost exactly similar to the result obtained by Tannenbaum (1947) when studying the effects of restricted diets on tumour genesis. His conclusions are summarized as a graph in Fig. 2, in which for comparison a graph of the present results is included. In both cases the form of the graph is sigmoid, and in both cases the maximum depression accompanies the reduction from 80 per cent to 70 per cent of the normal food intake. This adds further support to the thesis put forward by Bullough (1949*b*), and developed in the following review (Bullough, 1950*c*), that the rate of tumour genesis in a particular tissue or in the body as a whole may be directly related to the mitosis rate. The mitosis rate in turn is related to a variety of factors, of which one of the most important is the carbohydrate, or calorie, content of the food.

SUMMARY.

Groups of adult male mice were maintained for 4 weeks on diets varying from 55 per cent to 105 per cent of what they would eat if fed *ad libitum.*

Weekly records were made of the body weights and epidermal mitosis rates, and at the end of the experiments the carbohydrate reserves were estimated in terms of liver glycogen content and blood-sugar concentration.

It was found that body weight and carbohydrate reserve varied in direct proportion to the amount of food given. However, the mitosis rate did not vary directly in this way, and its relation to food intake can be expressed by means of a sigmoid curve. The greatest mitosis depression accompanied a reduction from 80 per cent to 70 per cent of the normal diet, a result which compares closely with that of Tannenbaum (1947) regarding the effects of restricted diets on carcinogenesis.

REFERENCES.

BULLOUGH, W. S.—(1948) *Proc. Roy. Soc. Lond.*, B, **135**, 212.—(1949*a*) *J. exp. Biol.*, **26**, 83.—(1949*b*) *Brit. J. Cancer*, **3**, 275.—(1949*c*) *J. exp. Biol.*, **26**, 287.—(1950*a*) *J. Endocrinol.*, **6**, 350.—(1950*b*) *Exp. Cell Res.*, **1**, 497.—(1950*c*) *Brit. J. Cancer*, **4**, 329.

*Idem* AND EISA, E. A.—(1950) *J. exp. Biol.* (in press).

GOOD, C. A., KRAMER, H., AND SOMOGYI, M.—(1933) *J. Biol. Chem.*, **100**, 485.

SIMPSON, G. G., AND ROE, A.—(1939) 'Quantitative Zoology.' London (McGraw Hill).

SOMOGYI, M.—(1930) *J. Biol. Chem.*, **86**, 655.

STEEDMAN, H. F.—(1947) *Quart. J. micr. Sci.*, **88**, 123.

TANNENBAUM, A.—(1940*a*) *Arch. Path.*, **30**, 509.—(1940*b*) *Amer. J. Cancer*, **38**, 335. (1942) *Cancer Res.*, **2**, 460.—(1944) *Ibid.*, **4**, 673.—(1945) *Ibid.*, **5**, 609.—(1947) 'Approaches to Tumor Chemotherapy.' Washington (Amer. Ass. Adv. Sci.).